# VACTERL (vertebral anomalies, anal atresia or imperforate anus, cardiac anomalies, tracheoesophageal fistula, renal and limb defect) spectrum presenting with portal hypertension: a case report

**DOI:** 10.1186/1752-1947-4-128

**Published:** 2010-05-05

**Authors:** Dilli Raj Bhurtel, Ignatius Losa

**Affiliations:** 1 Addenbrooke's Hospital, Cambridge University NHS Trust, Hills Road, Cambridge CB2 0QQ, UK; 2 Macclesfield District General Hospital, Macclesfield, UK

## Abstract

**Introduction:**

We report for the first time a unique case of VACTERL (vertebral anomalies, anal atresia or imperforate anus, cardiac anomalies, tracheoesophageal fistula, renal and limb defect) spectrum associated with portal hypertension. The occurrence of both VACTERL spectrum and extrahepatic portal hypertension in a patient has not been reported in the literature. We examined whether or not there was any association between extrahepatic portal hypertension and VACTERL spectrum.

**Case Presentation:**

A two-and-half-year-old Caucasian girl with VACTERL spectrum presented with hematemesis and abdominal distension. She had *caput medusae*, ascites, splenomegaly, gastric and esophageal varices. Her liver function tests were within normal limits. Magnetic resonance imaging of the liver with contrast showed a thready portal vein with collateral vessels involving both right and left portal veins without intrahepatic duct dilation.

**Conclusion:**

A thready portal vein, with features of extrahepatic portal hypertension, is a rare non- VACTERL-type defect in patients with VACTERL spectrum. Understandably, clinicians should give low priority to looking for portal hypertension in VACTERL spectrum patients presenting with gastrointestinal bleeding. However before routinely looking for a thready portal vein and/or extrahepatic portal hypertension in asymptomatic VACTERL spectrum patients, we need further evidence to support this rare association.

## Introduction

The clinical manifestation of VACTERL association includes vertebral anomalies, anal atresia, congenital heart disease, tracheoesophageal fistula, renal dysplasia and limb abnormalities [1]. Portal hypertension results from the elevation of portal venous pressure. The late consequences of portal hypertension may be esophageal varices, gastric varices, splenomegaly, ascites, and *caput medusae *[2]. The association of VACTERL spectrum and portal hypertension in a child has not been reported so far. We report a case of VACTERL association with portal hypertension and discuss the possibility of a common etiology.

## Case presentation

A two-and-half-year-old Caucasian girl presented with hematemesis. A systemic inquiry revealed no other symptoms. She was noted to be very small, with growth below the 3rd centile. She was pale, very alert, active and playful. Her abdominal examination revealed prominent superficial veins with splenomegaly measuring 6 cm from the costal margin. The rest of her systemic examination was normal.

Her stool was positive for occult blood; however her complete blood count, coagulation profile, extended clotting study, and thrombophilia screen were all within normal limits. She had had a Meckel's abdominal scan which came back normal. Her liver function which was repeatedly checked remained within normal limits.

Our patient was born by emergency lower segment Cesarean section (EmLSCS) due to fetal distress at 33 weeks' gestation. Her parents were non-consanguineous Caucasians. At birth her mother was 34 years of age, a non-smoker and a non-alcoholic; and she was on prophylthyouracil for a hyperactive thyroid. Antenatal history revealed that her pregnancy had been complicated by pregnancy-induced hypertension. Fetal growth had been monitored regularly for suspected growth restriction. Ultrasound scan had confirmed the restricted growth and a Doppler ultrasound scan had been abnormal with absent end diastolic flow. There had been no oligo- or polyhydramnios. Antenatal TORCH (toxoplasma, rubella, cytomegalovirus, Herpes Simplex), HIV, Treponemma and Hepatitis screening were all normal.

Our patient was born in a good condition with Apgars of 9 at one minute and 10 at five minutes. Her weight was 1250 g at birth, which was below the 3rd centile for her age and sex. She was noted to have an imperforate anus, with a rectovaginal fistula. She had heart murmur, and a subsequent echocardiography confirmed perimembranous ventricular septal defect (VSD) and persistent foramen ovale. An X-ray of her spine confirmed the hemivertebrae on her sacrococcygeal spine. She had had surgical correction of her imperforate anus and rectovaginal fistula within the first few days of life.

She had spent four weeks in the neonatal unit before being discharged home. She was doing well at home until she presented to us at two and half years of age with effortless vomiting of blood. Her growth trajectory had always remained below the 3rd centile.

She had had different tests following her admission with hematemesis. An ultrasound of her abdomen confirmed splenomegaly, and a Doppler sonography showed a thready portal vein with correct directional low velocity flow towards the liver.

**Figure 1:**
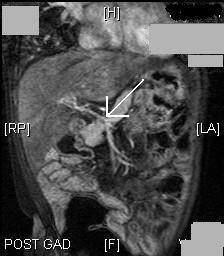
**Coronal section of the gadolinium-enhanced MRI scan of the liver and portal system**. The image shows a thready portal vein (arrow) with collateral vessels involving both left and right portal veins.

**Figure 2:**
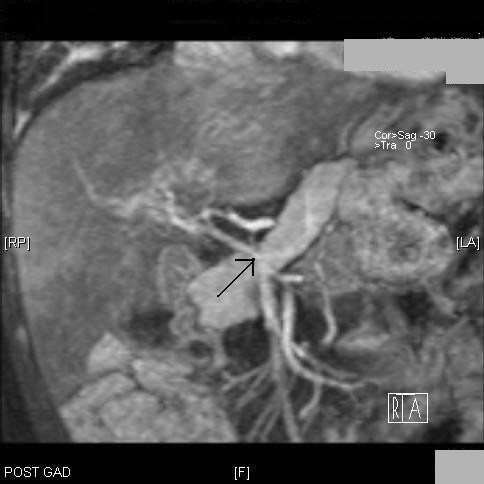
**Thready portal vein with prominent collaterals seen in the coronal section of the gadolinium-enhanced MRI scan**.

Magnetic resonance imaging (MRI) of her liver, using a gadolinium contrast agent, confirmed splenomegaly with gastric, retroperitoneal and splenic varices. The portal vein was thready (Figures 1 and 2) with collateral vessels involving both left and right portal veins. There was no evidence of intrahepatic duct dilation in the gadolinium-enhanced MRI scan of the liver.

## Discussion

Our patient had an imperforate anus with a rectovaginal fistula, perimemebranous VSD, persistent foramen ovale and vertebral anomalies; features associated with a VACTERL spectrum. She also had splenomegaly, gastric, retroperitoneal and splenic varices, along with dilated superficial abdominal veins which were the late seqelae of portal hypertension. MRI of the liver with contrast showed a thready portal vein with collateral vessels involving both right and left portal veins without intrahepatic duct dilation.

Non-VACTERL-type defects like single umbilical artery, genital defects and respiratory tract anomalies have been frequently described in patients with VACTERL association [3]. De Jong EM *et al*. stated that 70% of cases with VACTERL spectrum had additional non-VACTERL-type defects, with high occurrences of single umbilical artery (20%), genital defects (23.3%) and respiratory tract anomalies (13.3%).

Extrahepatic portal hypertension in children with normal liver function is not especially uncommon. The most common cause is portal vein thrombosis (PVT). Portal hypertension in our patient was noticed at two and half years of age when she presented to us with hematemesis. She was investigated for a possible etiology. Her thrombophilia screen and liver function tests were normal. Her imaging of the liver and portal system showed a thready portal vein with collaterals arising from the right and left portal veins.

Ando *et al*. studied portal venous anatomy in 10 patients with extrahepatic portal vein obstruction without hepatic disturbances ranging in age from one to seven years (mean age, 4.2 years) using ultrasonography, portal venography, computed tomography cholangiography and MRI [4]. The extrahepatic portal vein was not obliterated, but it crossed over the common bile duct from the left to the right side at the cranial level of the pancreas and ran in a cranial direction along the right side of the common bile duct or coiled itself around the bile duct. Thus, the extrahepatic portal vein formed a tortuous eta-shape. The portal vein was not obstructed in patients with extrahepatic portal vein obstruction but formed a characteristic eta-shape by coiling itself around the common bile duct, suggesting that extrahepatic portal vein obstruction has an embryological cause. We postulate that the thready portal vein seen in our patient could be another structural defect which had led to portal hypertension.

No definite gene has been identified to explain the VACTERL association and the etiology is not yet confirmed [5]. However during early embryonic development, disruption occurs, leading to different malformations of the heart, skeleton, muscle and blood vessels of the VACTERL spectrum. The disruption occurs in the differentiation of the mesoderm leading to the different malformations of the VACTERL spectrum.

It will be very difficult to derive any conclusion from a single case however it is worth looking at the possibility of future studies of VACTERL spectrum patients.

## Conclusion

Our case is the first of case of its type with VACTERL spectrum and extrahepatic portal hypertension. A thready portal vein, with features of extrahepatic portal hypertension, may be one of the non-VACTERL-type defects in patients with VACTERL association. But before actively looking for a thready portal vein and or extrahepatic portal hypertension in VACTERL spectrum patients, we need more evidence to support this hypothesis.

## Competing interests

The authors declare that they have no competing interests.

## Authors' contributions

DRB and IL both identified the case and prepared it. DRB collected the detailed information about the presentation of the case. All the investigations were collected and individually reviewed by DRB. MRIs were collected from St James Hospital. IL provided the detail information of the antenatal and significant past medical history. Both authors read and approved the final manuscript.

## Consent

Written informed consent was obtained from the patient's next-of-kin for publication of this case report and any accompanying images. A copy of the written consent is available for review by the Editor-in-Chief of this journal.
